# P-898. Reproducible profiling of eight Brazilian infectious diseases specialist training sites through open databases

**DOI:** 10.1093/ofid/ofae631.1089

**Published:** 2025-01-29

**Authors:** Guilherme Lira, Victor Ota, Yasmin Calzolari, Isabela Leitão

**Affiliations:** Universidade Federal do Rio de Janeiro, Rio de Janeiro, Rio de Janeiro, Brazil; Universidade Federal do Rio de Janeiro, Rio de Janeiro, Rio de Janeiro, Brazil; Universidade Federal do Rio de Janeiro, Rio de Janeiro, Rio de Janeiro, Brazil; Universidade Federal do Rio de Janeiro, Rio de Janeiro, Rio de Janeiro, Brazil

## Abstract

**Background:**

Many factors influence the choice of a specialty and a residency training site after essential medical education, including hospital characteristics. We set out to explore characteristics of infectious diseases (ID) and pediatric ID training sites in one Brazilian state, aiming to provide a data-driven input into residency site choice.

Number of hospitalizations and age and sex of patients

**Figure:**
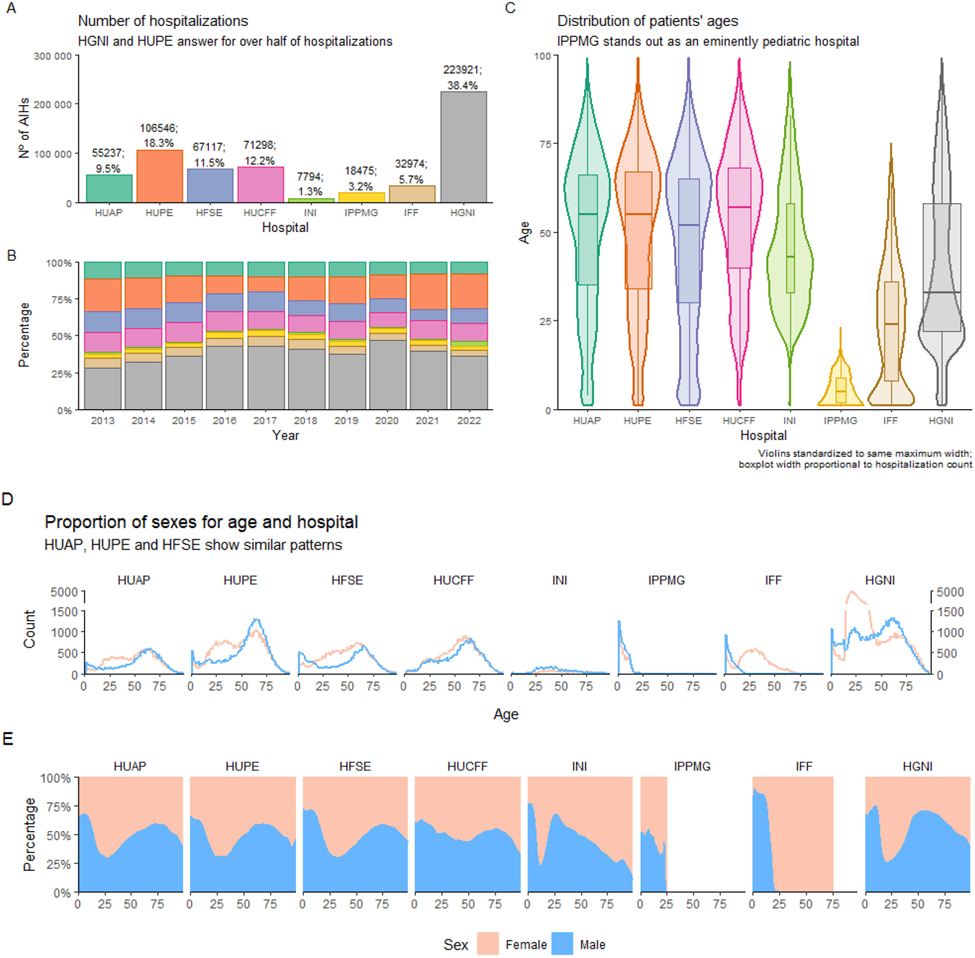

(A) Absolute number of hospitalizations per institution in the 10-year period, and (B) proportion of hospitalizations in each institution along the years. (C) Violin plots of ages for each institution with the same maximum width, and inner boxplots with width proportional to hospitalization count. (D) Frequency plots of male and female patients hospitalized in each institution, with (E) corresponding area plots adding to 100% for every age.

**Methods:**

We performed a retrospective, descriptive study of hospitalizations in institutions which offer ID and/or peds ID medical training, using Ministry of Health openly available hospitalization databases as sources. We used R software version 4.3.1 for analysis to ensure reproducibility.

Procedures performed, length of hospital stay and intensive care unit costs per institution

**Figure:**
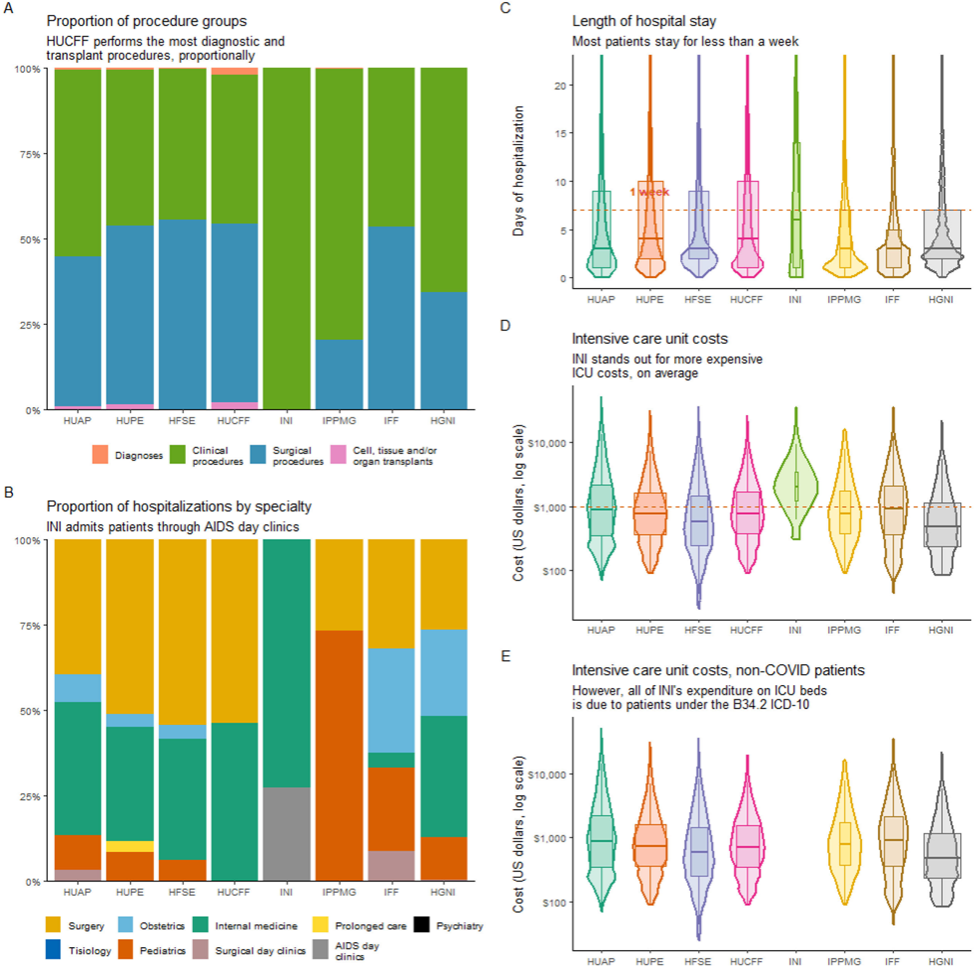

(A) Proportion of diagnostic, clinical, surgical and transplant procedures carried out in each hospital. (B) Proportion of specialty associated with hospital bed used for each hospitalization. (C) Violin plots of days of hospital stay for each institution. (D) Violin plots of total ICU costs (US dollars, log scale) in each hospitalization for each hospital; (E) the same plot, after removing COVID hospitalizations.

**Results:**

Eight institutions from Rio de Janeiro (RJ) fit the criteria and had available data. From January 2013 to December 2022, they carried out 583,362 hospitalizations. Two hospitals (HGNI, HUPE) accounted for 56.6% of these. The hospitals with peds ID positions (IPPMG, IFF) have different age distributions because one also admits birthing mothers. Most admissions were due to clinical or surgical procedures, with a minority cataloged as diagnostic procedures or cell, tissue and organ transplants. Most patients were discharged within 7 days. HUCFF had the highest proportion of diagnostic and transplant procedures (1.88% and 2.12%, respectively). On average, university hospitals are paid 2∼3 times as much as other institutions per hospitalization. Overall disease burden was diverse, but INI stood out as a center for HIV care.

Proportions of ICD-10 chapters or subchapters as leading causes of hospitalization

**Figure:**
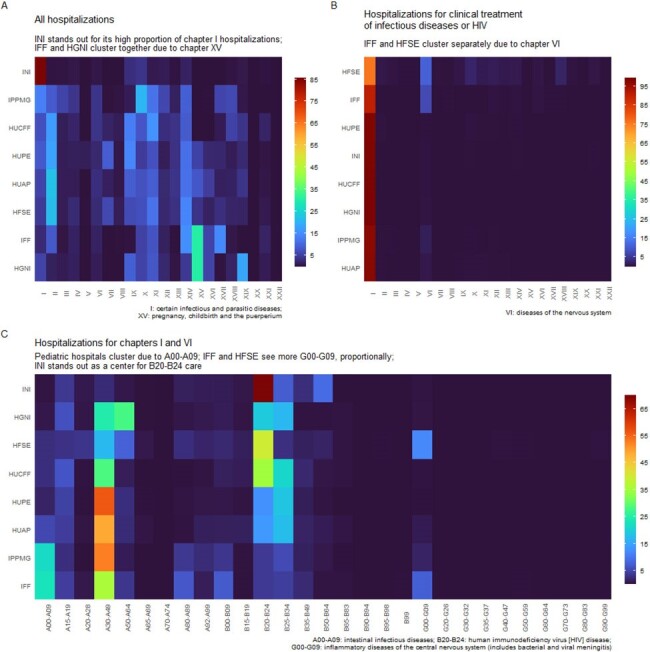

Heatmaps with unsupervised clustering of ICD-10 chapter or subchapter frequency as leading cause of hospitalization, for all hospitalizations (A), for hospitalizations filed under “clinical treatment of infectious and parasitic diseases” or “clinical treatment of HIV/AIDS” (B), or for hospitalizations filed under ICD-10 chapters I or VI (C).

**Conclusion:**

Hospitals in RJ with ID and peds ID residency spots have distinct workloads, patient profiles, disease burden and government-paid income. Residents from these programs might express similar views, but they do not necessarily reflect the widest picture, and might not be accessible to all interested candidates. Although subjectivity should not be discarded when choosing a training site, open data can inform more precise, transparent distinctions. For example, candidates interested in transplant ID could be more inclined to choose HUCFF, while candidates interested in less complex care could opt for HGNI. The framework we provide can also be employed to analyze other programs and hospitals, such as surgical residencies. Not all hospitals with residency positions feed these databases, thus limiting our analysis.

**Disclosures:**

**All Authors**: No reported disclosures

